# Cervical oesophagostomy in patients with severe dysphagia following radiotherapy for nasopharyngeal carcinoma

**DOI:** 10.1017/S0022215113003423

**Published:** 2014-01-28

**Authors:** Y-J Wang, W-X Chen, J-L Zhang, F-Y He, Z-F Zhu, Y Zeng, F Yang, S-C Tang

**Affiliations:** Department of Otorhinolaryngology, First People's Hospital of Foshan, People's Republic of China

**Keywords:** Nasopharyngeal Carcinoma, Dysphagia, Cervical Esophagostomy

## Abstract

**Objective:**

This study aimed to investigate the validity and feasibility of cervical oesophagostomy as a treatment for patients with severe dysphagia after radiotherapy for nasopharyngeal carcinoma.

**Methods:**

The study retrospectively analysed the clinical data, symptoms, physical signs, treatment and outcomes of 12 patients treated with cervical oesophagostomy for severe dysphagia after radiotherapy for nasopharyngeal carcinoma, from 2006 to 2010.

**Results:**

In all 12 cases, the oesophageal stoma remained stable, without any complications such as pharyngeal fistula or inflammation. No oesophageal stricture or granuloma growth was observed. All patients reported significant improvement in their nutritional status and quality of life after the oesophagostomy surgery.

**Conclusion:**

Cervical oesophagostomy is a valid and feasible method of treating severe dysphagia following radiotherapy for nasopharyngeal carcinoma. Oesophagostomy shows specific advantages over nasogastric tubing, gastrostomy and jejunostomy. Patients' nutrition and quality of life can be improved significantly if cervical oesophagostomy is executed in a timely fashion, especially in cases with severe trismus and multiple radiation-induced cranial nerve palsies unresponsive to rehabilitation.

## Introduction

The highest incidence of nasopharyngeal carcinoma (NPC) is found among Southern Chinese.^1^ Radiotherapy (RT) is currently the main and most effective treatment for this condition. In recent years, the survival rate of NPC patients has increased owing to developments in radiobiology and radiophysics and the clinical application of combination therapy (e.g. chemoradiotherapy). However, adverse radiation-induced effects, such as osteoradionecrosis, otitis media, nasosinusitis, cervical cutaneous necrosis and cranial nerve palsy, have gained greater recognition as survival times have increased.^2^

Radiation-induced dysphagia is one of the most common and serious long-term complications of RT. Many affected patients suffer from trismus, hoarseness, deglutitive cough and aspiration pneumonia, and some die due to pneumonia or cachexia.^3^^–^^5^

The present study assessed 12 patients, treated between 2006 and 2010, who had received RT for NPC but had developed severe dysphagia. They had subsequently undergone cervical oesophagostomy, and reported significant improvement in their nutritional status as well as quality of life as a result.

## Materials and methods

### Patients

Twelve patients with nasopharyngeal carcinoma (NPC) (9 men and 3 women; mean age, 54 years; age range, 32–70 years) received 1 or 2 courses of RT with curative intent. These patients' demographic and clinical information is summarised in Table I. The median RT dose delivered to the nasopharynx and the upper neck was 76 Gy (range, 65–98 Gy) and 63 Gy (range, 54–70 Gy), respectively.

**Table I:** Patients' demographic and clinical data

Pt no	Gender	Age (yr)	NP/neck RT dose (Gy)	Latency* (mth)	Inter-incisor distance (cm)	CN palsy	Pretreatment
1	F	32	69.0/56.0	3	2.5	–	Gastrostomy
2	M	44	77.6/61.2	108	2.0	IX, X, XI, XII	NG tubing, tracheostomy
3	F	70	70.0/66.0	60	1.0	IX, X, XI, XII	–
4	F	58	98.0/63.0	84	0.5	IX, X, XI, XII	Tracheostomy, gastrostomy
5	M	45	79.2/69.0	91	1.0	IX, X, XI, XII	NG tubing
6	M	59	81.0/70.0	72	0.7	IX, X, XI, XII	NG tubing
7	M	52	65.0/54.0	71	1.0	IX, XI, XII	NG tubing
8	M	66	80.0/64.0	187	3.0	IX, X, XI, XII	–
9	M	56	76.0/65.0	78	0.2	IX, X, XI, XII	NG tubing
10	M	55	71.0/61.3	93	0.2	IX, X, XI, XII	NG tubing
11	M	54	77.0/68.0	192	2.0	IX, X, XI, XII	NG tubing
12	M	40	68.2/58.5	98	1.0	IX, X, XI, XII	NG tubing

*Time between completion of radiotherapy (RT) and onset of dysphagia. Pt no = patient number; yr = years; NP = nasopharyngeal; RT = radiotherapy; mth = months; CN = cranial nerve; F = female; M = male; NG = nasogastric

Subsequently, all patients suffered severe dysphagia, with a median latent period of 8 years (range, 0.25–16 years), and almost all received surgery a mean of 13 months after this symptom appeared (range, 3–72 months).

The exception was one patient who underwent treatment earlier because of a wide ulcer which spread over the hypopharynx and oesophagus and resulted in complete stricture of the oesophageal entrance, five months after completion of chemoradiotherapy.

This latter patient also had a 1-cm hard palate fistula, but no cranial nerve palsy. The other 11 patients presented with cranial nerve palsies of varying degrees of severity involving the IXth, XIth, XIIth and usually also Xth nerves. Overall, there were nine patients with bilateral hypoglossal nerve palsy, of whom five had bilateral vagus palsy and four had unilateral vagus palsy; two cases required tracheotomy. The remaining two patients developed unilateral hypoglossal nerve palsy, in addition to unilateral vagus palsy.

Trismus (with an inter-incisor distance of 1 cm or less) occurred in eight patients owing to fibrosis of the masticatory muscles.

The 12 patients' main clinical manifestations were hoarseness, deglutitive cough, nasal regurgitation, difficulty with deglutition and recurrent aspiration pneumonia.

Their mean pre-oesophagostomy weight was 46.5 kg (range, 38–56 kg). Prior to this surgery, 8 patients had relied on nasogastric tube feeding for an extended period (range, 2–18 months). Seven of these eight patients had suffered nasal haemorrhage. Of the remaining four patients, two underwent gastrostomy six months and four years after RT, variously. The other 2 patients were capable of oral intake albeit with a prolonged meal time (range, 1.5–2.5 hours), complicated by easy choking and nasal regurgitation (Table I).

In all patients, the first step was to exclude the possibility of recurrence and metastasis, using mainly nasopharyngoscopy, magnetic resonance imaging or computed tomography, with biopsy if necessary. Patients with a history of gastroesphageal cancer were excluded.

Patients also underwent contrast oesophagography and fibre-optic endoscopic evaluation of swallowing to help evaluate and classify their dysphagia. The diatrizoate meglumine swallow sign indicated the absence of an oesophageal space-occupying lesion, except in one case of severe oesophageal stricture. Laryngeal penetration or aspiration was found in every case. All patients but one showed prolonged oral transit and difficult transport of the bolus to the pharynx, during fibre-optic endoscopic evaluation of swallowing, especially those patients with bilateral hypoglossal nerve palsy, resulting in pooling of contrast in the pyriform sinus and subsequent laryngeal penetration or aspiration.

### Methods

#### Anaesthesia

Patients who were post-tracheostomy or who had mild restriction of mouth-opening were operated upon under general endotracheal anaesthesia. Other patients required intravenous general anaesthesia or anaesthesia involving cervical plexus blockade.

#### Surgery

**Fig. 1:**
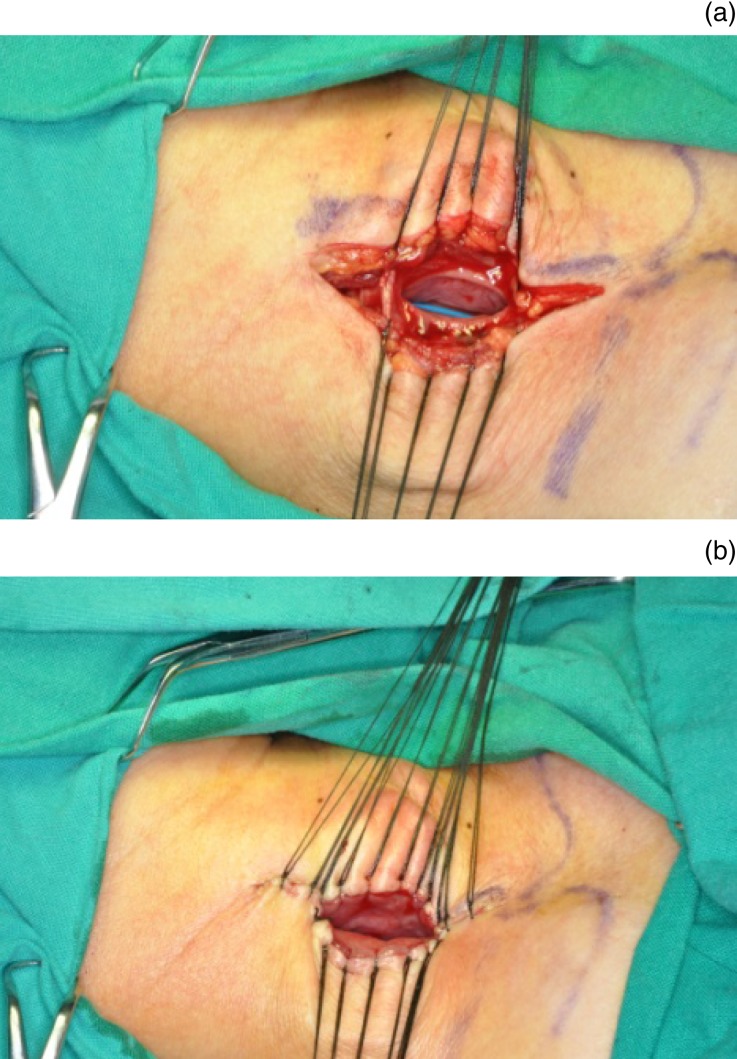
Surgical photographs showing cervical incision and creation of cervical oesophageal stoma. (a) A 2 cm, vertical length of oesophageal wall is excised from the divided oesophageal segment. (b) The two muscle layers of the oesophagus are sutured to the platysma muscle, and the oesophageal mucous membranes are sutured with the skin of incision.

A transcervical incision on the left side was chosen because the cervical oesophagus usually curves slightly to the left. A right-sided incision was performed in cases of ipsilateral vagus palsy with normal contralateral vagus function. A diagonal incision was made along the anterior border of the sternocleidomastoid muscle, through the skin and platysma muscle, proceeding along a line extending from the sternoclavicular joint to a point 1 cm cephalid to the cricoid cartilage. The sternocleidomastoid muscle was divided without exposing the carotid sheath, and the strap muscles were divided vertically to expose the thyroid. The thyroid was divided and temporarily elevated aside, following division and ligation of the middle thyroid vein at the lateral border of the gland. The cervical oesophagus was dissected for 2–3 cm in a superior and lateral direction, with identification and protection of the recurrent laryngeal nerve. In cases of severe constriction of the oesophageal entrance, the oesophagus was cut transversely below the stricture and sutured to the skin of the cervical incision. In cases without oesophageal stricture, the divided segment of the oesophagus was pulled out to the plane of the primary incision and a 2 cm length of oesophageal wall was vertically excised (Figure 1a). A 2.0 × 0.5 cm oesophageal stoma was created by suturing the two muscle layers of the oesophagus to the platysma muscle, and suturing the oesophageal mucous membranes to the incised skin (Figure 1b). Finally, the other incisions were closed in layers. The tracheotomy incision had little effect on the surgery as it did not connect with the oesophagostomy surgical incision.

## Results

**Fig. 2:**
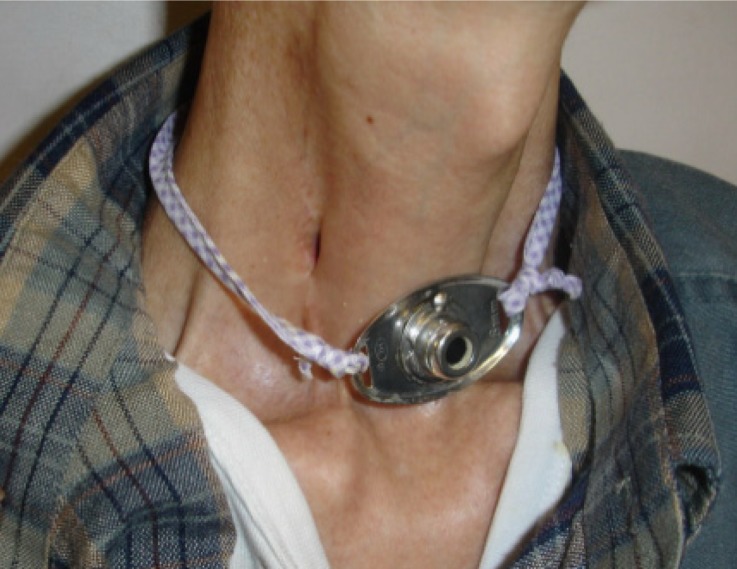
Clinical photograph showing the inconspicuous stoma remaining in the neck after cervical oesophagostomy.

**Fig. 3:**
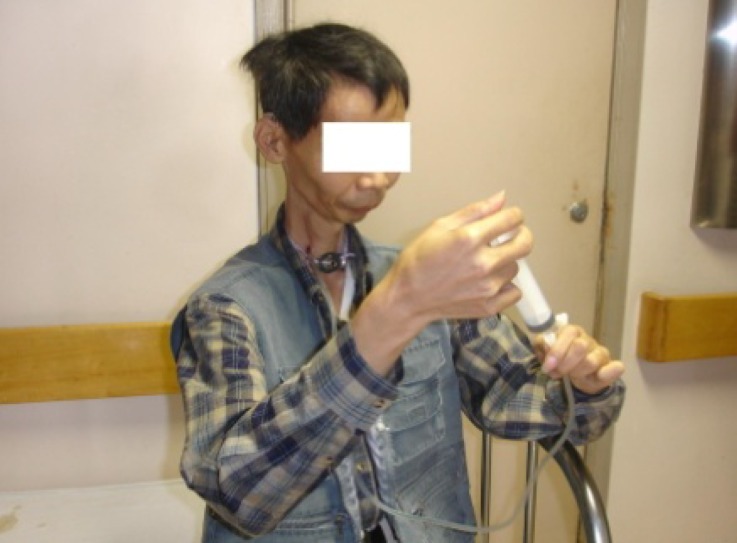
Clinical photograph showing a patient feeding himself through a tube inserted into his oesophageal stoma, following cervical oesophagostomy.

The 12 patients were followed up for a period ranging from 8 to 30 months after oesophagostomy surgery. In all cases, the oesophagostoma remained stable with a diameter of 6–7 mm (Figure 2). No case of post-operative oesophageal stricture or granuloma was encountered. At meal times, patient were able to insert a nasogastric tube (i.e. a flexible silicone tube with an inner diameter of either 16 or 18 mm) into their stomach through the cervical stoma. In this way, they could feed themselves fruit juice, soup and food crushed in a food blender (e.g. rice, vegetables, fish or meat) via the tube (Figure 3). After each meal, the tube was flushed with warm water and then withdrawn.

No complications were observed (e.g. pharyngeal fistula or inflammation). Where present, pre-operative tracheotomy had little effect on the oesophagostomy procedure or on post-operative feeding. Following oesophagostomy, no cases of aspiration pneumonia were encountered. Patients' nutritional status improved significantly after surgery, and their weight increased by an average of 5.6 kg (range, 4–7 kg). Furthermore, patients had only an inconspicuous neck stoma, and were reliant on a nasogastric tube only at meal times (for a time period ranging from 20 to 30 minutes). All patients reported improved satisfaction as regards appearance, convenience and comfort.

Over long-term follow up, two patients died of acute myocardial infarction and nasopharyngeal haemorrhage, variously, events which were independent of their cervical oesophagostomy.

## Discussion

Swallowing is one of the most complex human behaviours, and requires the precise co-ordination of over 40 pairs of muscles in the oral cavity, pharynx, larynx, oesophagus and neck, arising from neural control by the cranial nerves (i.e. Vth, VIIth, IXth, Xth and XIIth), sympathetic nerves and parasympathetic nerves. Normal swallowing can be divided into four phases: oral preparation, oral, pharyngeal and oesophageal.^6^

Several factors may contribute to the occurrence of dysphagia after radiotherapy (RT) for nasopharyngeal carcinoma (NPC).

First, radiation-induced fibrosis of the masticatory muscles and temporomandibular joint may result in trismus, which if severe may restrict or obstruct introduction of the food bolus into the mouth, making oral intake difficult or impossible.

Second, high irradiation doses can result from the overlap of RT fields (namely, the tangential low neck supraclavicular field, the pre-auricular field (used to target the carotid sheath), the accessorial anterior nasal and posterior auricular (brainstem) fields, and the cavernous sinus field), which may result in cranial nerve palsy affecting the IXth, Xth, XIth and XIIth nerves. Clinical features of glossopharyngeal and/or vagus nerve palsy include loss of the gag and palatal reflexes, poor palate movement on the side of the lesion, easy nasal regurgitation, deglutitive cough, hoarseness and achalasia of the cricopharyngeus muscle. Tongue deviation with atrophy signifies unilateral palsy of the hypoglossal nerve, while inability to protrude the tongue occurs with bilateral palsy. Palsy of any one of the cranial nerves mentioned above would probably lead to varying degrees of dysphagia; palsy of bilateral nerves, or of several different cranial nerves, is even more likely to cause this condition.

Last but not least, irradiation can hinder salivary gland function. The resultant reduction or absence of saliva impairs food bolus formation and oropharyngeal transit.

Hence, dysphagia after RT for NPC directly involves all four phases of swallowing. The occurrence of clinical features is affected by multiple factors. Patients with concomitant, bilateral hypoglossal, glossopharyngeal and vagus nerve palsies show prolonged oral transit, difficult transport of the bolus to the pharynx, poor gag reflex, weak muscle co-ordination and increased total pharyngeal transit time, which ultimately result in aspiration pneumonia and impaired nutrition. Severe dysphagia seriously affects patients' quality of life and is potentially life-threatening.

Dysphagia can be categorised as mild, moderate or severe.^7^ Mild dysphagia denotes a subjective complaint of difficulty in swallowing or occasional choking, which does not require dietary modification or swallowing therapy. Moderate dysphagia includes symptoms necessitating dietary modification or swallowing therapy. Severe dysphagia indicates a requirement for feeding tube placement. Moderate dysphagia can be relieved by such procedures as cervical oesophageal balloon dilatation,^8^ cricopharyngeal myotomy^9^^,^^10^ and laryngeal elevation.^11^ However, severe oropharyngeal dysphagia is difficult to ameliorate, even using laryngotracheal closure^12^ or laryngectomy; patients with severe trismus and bilateral hypoglossal nerve palsy are especially difficult to treat. In such cases, improvement of nutrition depends upon gastric tubing.

• This study assessed the effect of cervical oesophagostomy in nasopharyngeal carcinoma patients with severe dysphagia after radiotherapy
• No post-operative complication or aspiration pneumonia were seen
• Patients' nutritional status and weight improved significantly
• Cervical oesophagostomy was feasible and advantageous in these patients

Common approaches to enable gastric tubing include nasogastric tubing, gastrostomy, percutaneous gastrostomy and jejunostomy. Nasogastric tubing is relatively simple, convenient and cheap, but has the disadvantages of difficult insertion, easy displacement, image disturbance, subjective nasal and pharyngeal discomfort, problematic follow up, and the risk of pneumonia.^13^ Gastrostomy and percutaneous gastrostomy enable distinct improvement in patient nutrition, as a wider tube can be used; however, they also have significant negative effects on the patient's quality of life due to the abdominal stoma tube interfering with daily activities (e.g. bathing, dressing and transportation), and in addition pose a risk of local infection.^14^^,^^15^ Jejunostomy requires the use of a tube of smaller inner diameter, limiting the patient to a specific liquid diet; furthermore, the tube cannot remain in position long term, and replacement requires re-operation.

In contrast to the above approaches, cervical oesophagostomy is easy to perform, with minimal risk of injury. Patients have an inconspicuous stoma in the neck and are able to live independently of the feeding tube except for a short time during meals. A nasogastric tube is inserted into the stomach via the cervical stoma, injected with prepared food, and then withdrawn after finishing the meal. Using this simple procedure, patients can feed themselves with less discomfort and a lower risk of complications, and can enjoy an overall improved quality of life.

Overall, patients with severe dysphagia secondary to RT for NPC can have significantly better nutrition and quality of life if cervical oesophagostomy is executed as soon as possible.
